# A Doppler ultrasound self-monitoring approach for detection of relevant individual decompression stress in scuba diving

**DOI:** 10.1007/s11739-021-02802-z

**Published:** 2021-07-09

**Authors:** Andreas Fichtner, Benedikt P. Brunner, Thomas Pohl, Thomas Grab, Tobias Fieback, Thea Koch

**Affiliations:** 1 Head of Emergency Department and OR Management, Kreiskrankenhaus Freiberg, Donatsring 20, 09599 Freiberg, Germany; 2 University of Vienna, Vienna, Austria; 3 Scientific Diving Center, TU Bergakademie Freiberg, Freiberg, Germany; 4 Department of Anesthesiology and Intensive Care Medicine, University Hospital Carl Gustav Carus, Technische Universität Dresden, Dresden, Germany

**Keywords:** Decompression, Bubble detection, Scuba diving, Self-monitoring, Audio Doppler

## Abstract

Observing modern decompression protocols alone cannot fully prevent diving injuries especially in repetitive diving. Professional audio Doppler bubble measurements are not available to sports scuba divers. If those non-professionals were able to learn audio Doppler self-assessment for bubble grading, such skill could provide significant information on individual decisions with respect to diving safety. We taught audio Doppler self-assessment of subclavian and precordial probe position to 41 divers in a 45-min standardized, didactically optimized training. Assessment before and after air dives within sports diving limits was made through 684 audio Doppler measurements in dive-site conditions by both trained divers and a medical professional, plus additional 2D-echocardiography reference. In all dives (average maximum depth 22 m; dive time 44 min), 33% of all echocardiography measurements revealed bubbles. The specificity of audio bubble detection in combination of both detection sites was 95%, and sensitivity over all grades was 40%, increasing with higher bubble grades. Dive-site audio-Doppler-grading underestimated echo-derived bubble grades. Bubble detection sensitivity of audio Doppler self-assessments, compared to an experienced examiner, was 62% at subclavian and 73% at precordial position. 6 months after the training and 4.5 months after the last measurement, the achieved Doppler skill level remained stable. Audio Doppler self-assessment can be learned by non-professionals in a single teaching intervention. Despite accurate bubble grading is impossible in dive-site conditions, relevant high bubble grades can be detected by non-professionals. This qualitative information can be important in self-evaluating decompression stress and assessing measures for increased diving safety.

## Background

During ascent in scuba diving, inert gases such as nitrogen can become supersaturated in tissues and blood. This results in microbubbling and macrobubbling and ultimately can lead to symptoms of decompression sickness. To avoid this, decompression tables and dive computers provide empiric guidance on ascent time, depending on depth and dive time. However, asymptomatic inert gas bubbles arise frequently—up to the majority of air dives—even within sports diving limits [1], and there is a wide inter- and intraindividual variety in developing bubbles and decompression symptoms, despite following dive computer profiles that calculate ascent schedules from depth-time integrals. Dive depth, time and ascent speed are key factors for inert gas bubbling, although modified by individual factors, that can lead to so-called “undeserved” diving accidents despite following real time dive computer ascent protocols. In previous studies, a high number of detectable bubbles after ascent in up to 50% of divers was related to symptoms of decompression sickness in 2–11% of sports dives and up to around 40% of decompression and mixed gas commercial dives [1–3]. The risk for developing decompression-related symptoms was increasing significantly with an additional Odds ratio of 2.7 per bubble grade and a maximum risk of 43% to develop symptoms when bubbles are as frequently seen as at least 1 per cm^2^ in 2D echocardiography [3]. Symptomatic divers need oxygen and recompression treatment, however asymptomatic bubbling is not considered for treatment in sports diving and in most cases not diagnosed at all.

Audio Doppler ultrasound measurements comprise an established [2–8], validated [9] and standardized [10] method of monitoring the post-dive bubble load. Although semiautomatic computerized bubble quantification has been published [1, 11] and realized [12], it is still an exclusive skill of a medical or ultrasound professional, and is therefore not implemented in sports diving. Doppler monitoring of dives could contribute significantly to individual diving safety, and the technical devices required are of minor cost compared to scuba equipment itself.

However, unknown factors include the effort that is needed to train divers without medical or ultrasound expertise to allow them a sufficient self-assessment with audio Doppler, and how such results are correlated with results from an experienced sonographer and echocardiographic visual bubble detection as reference. Further, for efficiently implementing this skill in the broad sports diver community, the training must be a single intervention that guarantees a reliable and sustainable skill level, and the equipment price should be of a low purchase threshold.

The aims of the present study were to answer the following:

Can scuba divers, as non-professionals, be trained in a single teaching intervention to perform audio Doppler ultrasound for inert gas decompression bubble detection at the subclavian and precordial position? Endpoints: stable venous signal in less than 120 s.

What learning curve is required to generate reliable and consistent readings of a venous signal? Endpoints: no relevant improvement in time and failure rate.

Are the audio Doppler self-measurement results sufficient enough to determine reference bubble grades measured by 1. a medical and ultrasound professional by audio Doppler and 2. by a medical and ultrasound professional by visual 4-chamber echocardiography at dive-site conditions? Endpoints: correct qualitative bubble recognition. Correct bubble grading. Detection sensitivity and specificity.

## Methods

We recruited 41 scuba divers who took part in a Scientific Diver education course and undertook a total of 342 open-circuit air dives with not more than moderate exertion, mandatory safety stops and within decompression limits (no omitted safety and decompression stops), always with the obligation to follow wrist computer limits. There was a maximum of 2 dives per diver and day (morning and afternoon). Before and after single and repetitive dives, we recorded a total of 684 measurement sets with bubble self-recording via an 8 MHz audio Doppler ultrasound pencil probe (DopFlow, Spead Doppler Systems Germany) at both subclavian and precordial (left parasternal) sagittal position for optimized venous flow signal after a single standardized, 45-min session of theoretical and practical training before the measurements. All measurements were conducted in a mobile examination tent, adjacent to the dive site, 30 min before and after every dive. After undressing, the divers were placed in the same beach-chair position, and dive data (depth, time, decompression and safety stops, surface interval, individual stress and other events during the dive) were recorded. The divers were checked for any signs of diving injury by a trained physician. Later, wrist dive computer profiles were checked again for any signs of non-compliance during ascent (yo-yo-diving, ascent speed, omitted safety stops). Audio Doppler self-measurements were performed by the diver at both subclavian and precordial probe position and directly observed by an experienced examiner for a stable venous signal without artifacts, caused by the loss of an adequate ultrasound window. Subsequently, attempts were made to recognize High-Intensity Transient Signals (HITS)-like bubble signals within 1 min in the established venous signal. Times until a sufficient venous signal at both subclavian and precordial probe positions were recorded by experienced examiners. A required time of more than 120 s was considered to be insufficient and therefore an invalid measurement. Subsequently, the result of the diver’s interpretation of Spencer bubble grade was noted. These were compared with the same audio Doppler measurements made by an experienced examiner (ultrasound-trained medical professional) immediately thereafter, using the same ultrasound machine. Right after audio Doppler and without any gap, 30 s of representative 4-chamber echocardiographic loops were recorded using the same GE Logic e (General Electrics Healthcare, Solingen) ultrasound machine with a curved array multi-frequency probe and angulation through the heart by an experienced sonographer. All loops were later assessed again by two independent, experienced and blinded sonographers (advanced European ultrasound diploma). Detectable bubbling was recorded and graded using the Spencer Scale for audio Doppler assessments and the Eftedal–Brubakk (EB) scale for visual echocardiographic assessments [10], Table 1.

**Table 1:** Modified Spencer and Eftedal–Brubakk scales for audio Doppler and 2D echo bubble grading as adapted for our study. Both are categorized, non-linear scales, and a direct comparison of single grades is difficult

Modified Spencer scale for audio Doppler bubble detection	Bubble grades	Eftedal–Brubakk scale for echocardiographic bubble detection
No adequate signal	X	No adequate signal
No bubbles detectable	BG0	No bubbles visible
Occasional bubbles	BG1	Occasional bubbles
Bubble signals in less than half of heartbeats	BG2	At least 1 bubble/4 heartbeats
Bubble signals in most of heartbeats	BG3	At least 1 bubble/heartbeat
Bubble signals continuously and predominantly	BG4	At least 1 bubble at every cm2 in every view
	BG5	Whiteout—no single bubble discrimination

However, a rough relation of lower, medium and higher bubble grades between such scales and their different underlying measurements is considered adequate in this study

The audio recordings were made in a real dive-site environment without any surrounding noise reduction, the mobile examination tent sheltered against sun and rain only. The measurements were conducted over two weekends of scientific scuba training in a German freshwater lake (Ammelshain and Senftenberg) and two consecutive weeks of a diving expedition in seawater in Sveta Marina, Croatia. The study participants had not been diving for four weeks in between the measurement intervals and drove to Croatia with one day of rest before diving. We aimed at a single measurement time interval at peak bubble time of 30–40 min after each dive [13] for conducting our measurement sequence as described above. Additional repeated measurements after single dives to follow bubble development over time were not relevant to the present study.

Standardized training schedule:

1. Explanation of blood flow and established venous bubble detection sites, theoretical presentation—10 min.
2. Practical audiovisual demonstration of venous and arterial signals and Doppler angulation at both detection sites in human—10 min.
3. Explanation of the Spencer and Eftedal–Brubakk scales along with audiovisual simulation of each grade of this scale—5 min.
4. Guided self-examination at both subclavian and precordial detection site until a stable venous signal was established, using a modified Peyton’s Four-Step Approach [14] for complex skill teaching—20 min, including observation and participation of each other’s guided attempts at establishing individual anatomical ultrasound windows and interpreting simulated bubble grades.

Six months after initial training and 4.5 months after the last measurement without further practice, the divers were assessed again for their retention of audio Doppler self-assessment skills. Time until stable venous signal at both detection sites was measured by the same examiners to gather information about the long-term sustainability of the training intervention.

All divers signed informed consent forms, and the university ethics committee of the Technical University Bergakademie Freiberg approved the study plan. Data acquisition, storage and processing were performed after anonymization and following current ethical standards in sport science research. Depending on direct measurement results before and after any dive, the divers received safety information on surface interval and fluid intake. The study was supported by GTUEM e.V. (German Society for Diving and Hyperbaric Medicine) and General Electric’s Ultrasound division in Germany regarding material provisions.

Data analysis and presentation were conducted using R v4.0.3.

Experience at collecting measurements was defined as the total number of individual measurement cycles, (one subclavian measurement, plus one precordial measurement), conducted by each participant throughout the study, independently of the sampling occasion.

To estimate the effect of Doppler pen positioning, pre-dive and post-dive, and measurement experience, we utilized a linear mixed-effects model (LMER) with the participant as random-effect. The relation between the number of invalid measurements was also correlated with the experience using a Spearman correlation. The specificity and sensitivity of the self-measurements were calculated by comparing them to the examiner’s Doppler measurements. In addition, both were compared to reference echocardiography using Monte Carlo Chi-square test.

## Results

### Audio Doppler and echocardiography

The dives covered a broad spectrum of diving profiles and the dive time ranged from 4 to 83 min, *M* = 44.0, SD = 14.6 min; maximum depth ranging from 3 to 40 m, *M* = 21.8, SD = 9.5 m. In 28 of 342 dives, the dive computer indicated a single-level decompression stop at 3 m, which was observed in addition to the 3 min safety stop.

From a total of 684 reference echocardiographic measurements done by the same ultrasound professional, 224 measurements showed bubbles in the right atrium and ventricle and also in the inferior vena cava. EB grade distribution of bubble-positive measurements (*n*) was

EB0 *n* = 412, EB1 *n* = 136, EB2 *n* = 28, EB3 *n* = 33, EB4 *n* = 24, EB5 *n* = 3, n.a./invalid *n* = 48.

**Fig. 1:**
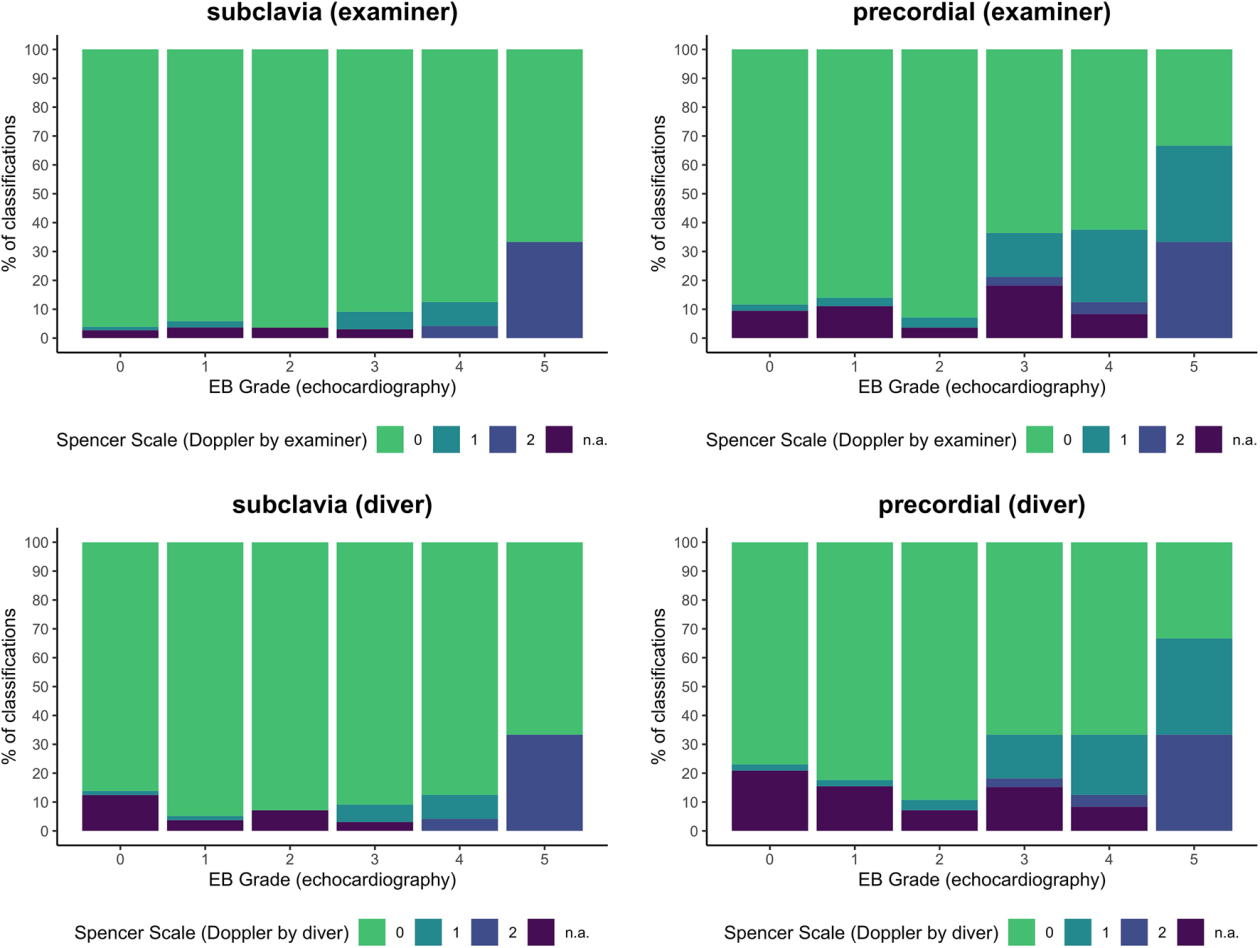
Echocardiography (EB grade) vs. Doppler measurements (Spencer grade) from reference measurements of an experienced medical professional (above) and the trained diver (below). Doppler associations to echocardiographic reference measurements are shown by percentage of audio Doppler detection (Spencer grade, examiner) of 100% of the respective EB grade number

The comparison of the audio Doppler-derived Spencer grades to reference echo-derived EB grades is shown in Fig. 1 for both diver and professional examiner. The EB grades, classified by echocardiography, are not resembled by the Spencer grades from Doppler measurements of either participants or examiners. However, there is a strong association between both grading results for subclavia (Monte Carlo Chi-square test, *p* < 0.001) and precordial (Monte Carlo Chi-square test, *p* < 0.001) measurements, despite the low measurement number of high EB grades.

In the case of EB grades > 1 after the first dive, lower EB grades were found frequently right before reentering the water for the second dive.

## Self-assessment

Defining the audio Doppler estimation of the examiner as the measurement standard in field conditions at the dive sites, the self-measurement in subclavian position showed a sensitivity of 61.5% and a specificity of 99.3%, compared to 72.7% sensitivity and 99.9% specificity in precordial position.

The participants completed on average of 15.0 (SD = 14.06, min = 1, max = 41) measurement cycles (“experience”). Some of the self-measurements were carried out in Germany (*M* = 7.2, SD = 4.2), and the majority in Croatia two months later (*M* = 13.8, SD = 9.9). Due to the small timespan in between, there was no further differentiation between those occasions.

**Fig. 2:**
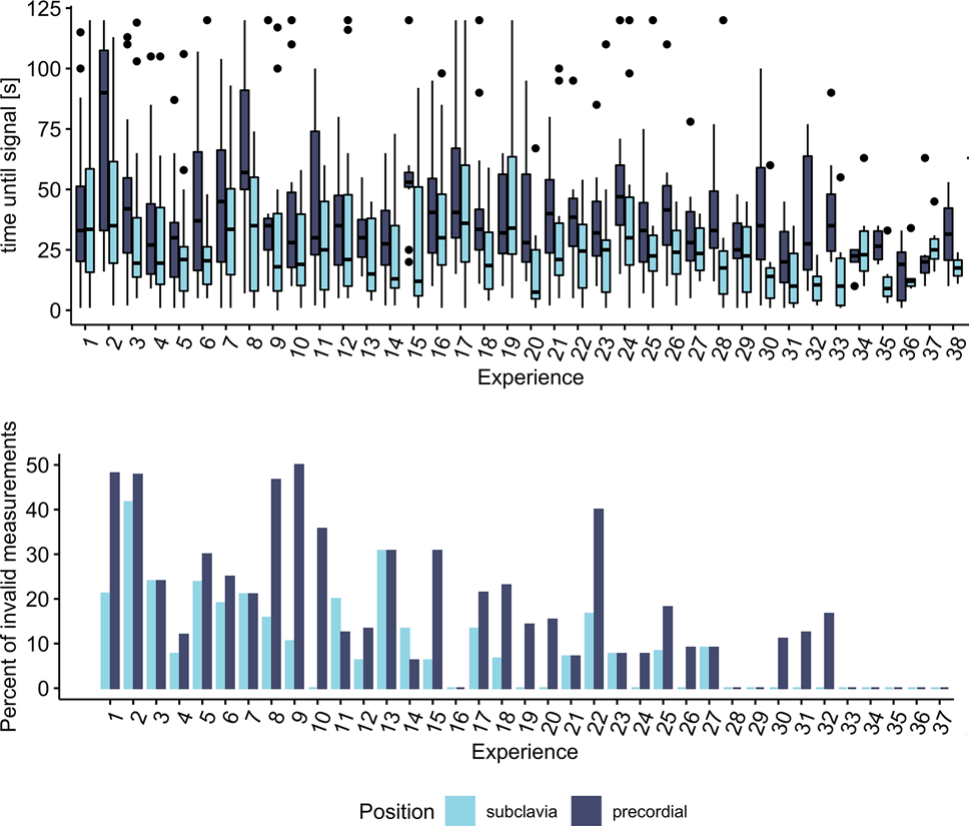
top: Time until signal by measurement cycles per participant (“Experience”). Bottom: Percentage of invalid measurements (the participant failed to retrieve a sufficient readable venous signal within 120 s) by experience. For subclavian measurements, mean time to signal is below 40 s after 18 attempts, compared to 25 attempts for precordial measurements. Failure rate is stable on a low level after 15 measurements in subclavian position and 25 in precordial position

The participants required an average of 38.9 s (SD = 28.2) to find a stable and readable venous signal without artifacts. With subclavian measurements (*M* = 29.5 s, SE = 1.61), the participants found the signal faster than with precordial (*M* = 40.7 s, SE = 1.61) measurements (*p* = 0.002, Table 2). Moreover, the time needed decreased 0.39 s when the participant gained (one) experience (Slope B =  − 0.39, SE = 0.09, *p* < 0.001, LMER, Fig. 2). The required time until sufficient venous audio signal did not differ depending on the time of measurement (before or after the dive, *p* = 0.448, Table 2). Furthermore, a low participant-specific effect was found (ICC = 0.03).

**Table 2:** Repeated measures analysis of variance (ANOVA) for time until signal [s] based on linear mixed-effects regression (LMER)

Source of variation	SS	DF1	DF2	*F*	*p*	Partial eta^2
Position (subclavia, precordial)	8271.3	1	826.4	11.29	0.001	0.01
Experience	14,215.8	1	524.0	19.41	< 0.001	0.03
Pre- or post-dive	449.6	1	845.1	0.61	0.434	0
Position × Experience	0.0	1	826.4	0.00	0.999	0

*ICC* 0.03 (intraclass correlation)

**Fig. 3:**
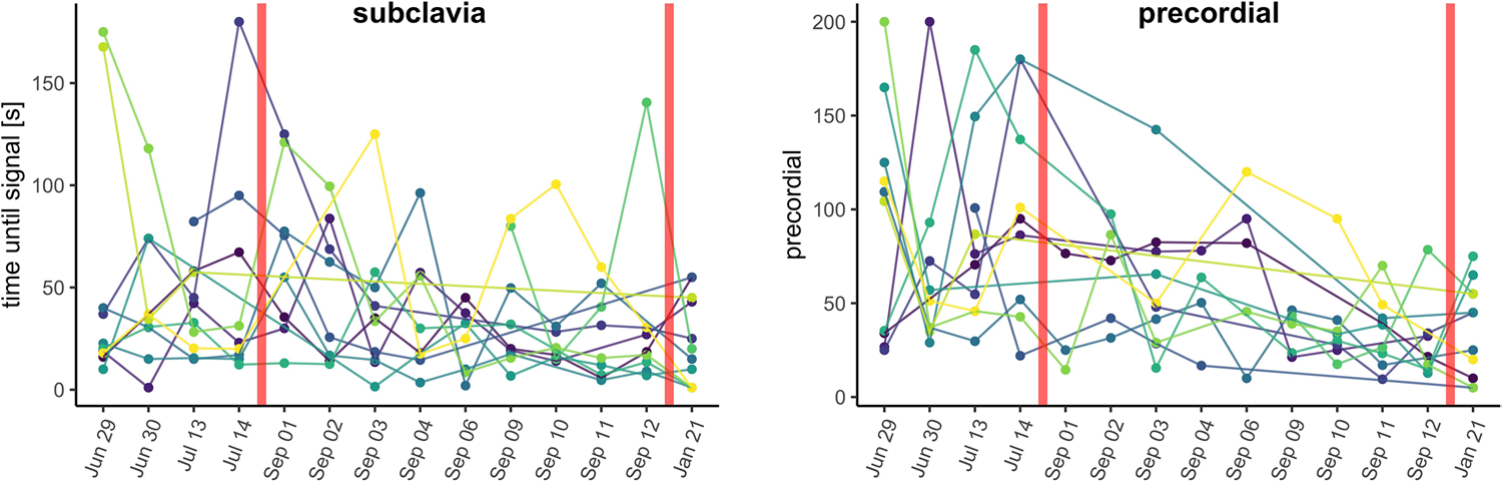
Individual learning curve of 12 participants (color) by date. Longer training gaps are annotated with a red line. A retention measurement was taken ~ 4.5 months after the last dive and more than 6 months after initial training

For subclavian measurements, the participants failed in 65 of 616 (10.5%) measurements to find a sufficient venous signal within 120 s and keep it stable. The proportion of invalid measurements was significantly higher for precordial measurements with 105 of 616 (17.0%; Chi-sq test, Chi-sq(1) = 10.92, *p* < 0.001). For both subclavian (r_s_ =  − 0.76, *p* < 0.001) and precordial (rs =  − 0.73, *p* < 0.001) measurements, a negative correlation of the invalid measurements with the participant’s experience was found (Fig. 3).

## Retention

The participants did not practice between mid-July and the beginning of September and again between end of September and January–however, there was no sign of individuals losing practice over longer training breaks. In the retention exercise 4.5 months after the last Doppler measurement and more than 6 months after initial training, the participants needed on average 20.6 (SD = 21.4) seconds for subclavian and 35 (SD = 25.5) seconds for precordial measurements. The time for the subclavian (paired *t* test, t (10) =  − 1.41, *p* = 0.188), along with the precordial (paired *t* test, t (9) =  − 0.65, *p* = 0.531) measurement in the retention exercise did not differ from the average time needed in the last four dives before the break.

## Discussion

Considering that our study divers followed standard sports diving profiles not exceeding moderate exhaustion, and did not omit safety stops, and that very few decompression stops were required, the observed percentage of divers with detectable bubbles in reference echocardiography was high and comparable to similar research (with a higher percentage after more provocative dive profiles [15, 16]).

If bubbles were detected during the first dive of the day, then it was common to detect residual bubbles immediately before divers re-entered the water for their second dives.

However, apart from fatigue, no symptoms that could be related to a decompression injury were recorded.

Due to evolving ultrasound technology and the recordings performed at the dive site without noise cancellation, we were able to show a clear advantage of 2D echocardiography for bubble detection in contrast to previous studies [16].

Our results show that sufficient audio Doppler bubble grading (Doppler measurement according to the Spencer scale and echocardiographic reference according to the EB scale) may be of limited compatibility if measured in a realistic field setting with background noise present. Both, divers and medical examiners were able to detect only higher bubble grades in audio Doppler but classified them as lower bubble grades compared to 2D echo reference. However, this is an important qualitative information on relevant bubbling and high decompression stress with a consecutively high risk of developing symptoms of a decompression accident. Further, low-grade bubbling and thus irrelevant decompression stress is not detected using audio Doppler in dive site conditions and in all gradings from audio Doppler, we only recognized underestimations but no overestimations of the reference echo-derived bubble grades. Therefore, and for practicability of dive site analysis, we suggest classifying the Doppler measurements into the two following categories: no relevant bubbles (single bubble signals, even if frequent = 0) and relevant bubbles present (continuous, predominant bubble signals, bubble showers = 1). These two categories may be equivalent to the bubble grades after EB 0, 1, 2, 3 and 4, 5.

Using this method, the Doppler measurement by the examiner would have a sensitivity of 14.8% and a specificity of 98.2% for subclavian position and would have a sensitivity of 36.0% and a specificity of 96.5% for precordial position compared to echocardiographic reference. For the self-measurement, this leads to a sensitivity of 14.8% and a specificity of 98.2% for subclavian position and would have a sensitivity of 32.0% and a specificity of 96.3% for precordial position compared to echocardiographic reference. Moreover, a combination of both measurements leads to a maximum dive-site sensitivity of 40.0% in our data (Table 3).

**Table 3:** Sensitivity and specificity of measurement methods within 95% confidence interval

Measurement	Reference	Sensitivity (%)	Specificity (%)
Doppler examiner subclavia	Echocardiography (adapted)	14.8 (4.2, 33.7)	98.2 (96.8, 99.1)
Doppler examiner precordial	Echocardiography (adapted)	36.0 (17.9, 57.5)	96.5 (94.6, 97.8)
Doppler examiner combination	Echocardiography (adapted)	40.0 (21.1; 61.3)	95.1 (93.0, 96.7)
Doppler self-subclavia	Echocardiography (adapted)	14.8 (4.2, 33.7)	98.3 (96.8, 99.2)
Doppler self-precordial	Echocardiography (adapted)	32.0 (14.9, 53.5)	96.3 (94.3, 97.8)
Doppler self-combination	Echocardiography (adapted)	36.0, (18.0, 57.5)	94.9 (92.6, 96.6)

Qualitative interpretation of relevant bubbling through classification of echocardiographic EB scale 0, 1, 2, 3, classified as 0, and EB scale 4, 5 as 1

Self-assessment at the precordial position seemed to be more difficult. This was mainly due to the prominent cardiac signals at this position. However, the detection sensitivity at this position was doubled compared to the subclavian position, probably due to the inclusion of venous bubble drainage from the lower body—which is especially relevant after extensive fin swimming while diving. This must not necessarily be contrary to previous findings of better bubble detection at the subclavian site [9] since it is easier to establish an adequate subclavian Doppler signal, as seen in our study as well. Further, in the precordial position, we have seen a higher amount of higher bubble grades in particular. When bubbles were detected through audio Doppler, the bubble grade was typically lower compared to the echocardiographic method, despite no significant time lag. The average difference in our study was two bubble grades lower in audio Doppler detection, compared to the echo reference bubble grade. Considering the number of measurements in our study, this difference can be relevant in predicting decompression outcomes [17]. Therefore, a classification of sports diver audio Doppler readings into only two categories would simplify the process for self-measurements at the dive site and could provide sufficient qualitative information on necessary safety precautions, such as increased surface interval, fluid intake, rest, avoiding flights or mountain drives, etc. Further, our study showed that trained examiners also had grading problems in dive-site conditions. Both participants and examiners were only able to recognize higher bubble grades in audio Doppler measurements in a mainly qualitative way.

Considering that bubbling occurs regularly in dives within sports diving limits and may be underestimated by diving algorithms and dive computer-derived ascent protocols due to a significant individual factor [13], any additional information on an increased or relevant individual decompression stress could add valuable information on recommended post-dive behavior for increasing diving safety. No matter what bubble grade, no diver considered the ultrasound result serious enough to seek medical attention. However, divers with self-detected bubbles were cautious for the next dive and increased fluid intake and avoided further extensive inert gas load through a safer dive profile in the following dive. Divers with high bubble grade in echo took adequate measures by increasing surface interval and/or skipping further diving that particular day.

A detection sensitivity of around 40% with a specificity of 95% in a field setting after a combined precordial and subclavian audio Doppler self-measurement, performed by the diver in about a minute, bears the potential of being further developed and/or enhanced by noise reduction and (semi-)automatic measurement using computerized algorithms [12].

### Skill retention

Six months after initial training, the previously achieved skill level was reliably preserved—a venous Doppler signal of adequate quality was self-detected within the same time compared to the end of a learning curve after initial teaching and individual anatomical ultrasound windows were remembered. This long-term skill retention of similar condensed 45-min standardized training using the modified Peyton method has already been shown in one study on teaching central line placement [18]. Hence, the training proved to be suitable enough to generate a stable, practical skill level for establishing a venous Doppler signal over time.

## Limitations

A limitation in generalizing our results might be that we used an 8 MHz probe instead of the more commonly used 4 MHz probe for such assessments. This might influence results, especially in heavier divers. Starting the pretests of this study with a 4 MHz pencil probe—since low-frequency Doppler examinations of divers are described for reliable signals—we recognized only minor challenges in detecting a sufficient venous signal in our mainly slim study population (average BMI *M* = 25.7, SD = 3.7, ranging from 20.1 to 33.8), even if attempts were made with several (handheld and portable desk) audio Doppler machines with a purchase price of as low as a few hundred EUR each. Both venous signals and bubble signals as HITS were detected much better with an 8 MHz pencil probe, which was then chosen for all our Doppler recordings.

Our interpretations were based on a comparable low number of dives with high bubble grades; therefore, the sensitivity of audio Doppler self-detection could be underestimated, as we only had three times EB grade 5 in our dataset. Further, since audio Doppler and echocardiographic measurements—despite very short time difference and within bubble peak—were performed serially, not simultaneously, bubbles might not have been present in either of the measurements to the same extent. However, we did not notice any changes in EB grades during the few minutes of sequential audio Doppler and 2D-echo examinations in our pretests. On the other hand, we wanted to retrieve information on practicability of audio Doppler measurements at dive-site conditions without noise reduction and possible distraction. The dive-site setting seems to be a relevant confounding factor, causing a lower sensitivity and only providing qualitative information on bubbles, which does not allow for adequate audio grading. However, this qualitative information, derived around peak bubble time, might be sufficient and simple enough to provide additional value to a sports diver without a professional medical or ultrasonographical background.

## Conclusion

Scuba divers without medical or ultrasound expertise are able to learn audio Doppler self-assessment and generate qualitative results. Doppler skills of retrieving a sufficient and readable venous signal can be reliably learned within 45 min of focused standardized teaching and very limited practical training, which enable stable results even after months without practice. Dive-site conditions allow a high specificity, but only a moderate sensitivity for Point-of-Care audio Doppler bubble recognition compared to 2D echo reference and only relevant high bubble grades are detected, whereas low bubble grades are missed. This qualitative information is crucial to the diver since it suggests the necessity of safety measures to avoid further increase of inert gas bubbling and finally, decompression-related symptoms. Therefore, qualitative audio Doppler self-detection could be further evaluated for inclusion in advanced scuba diver education under standardized conditions.

## Data Availability

The source data are available on request.

## Abbreviations

Scuba: Self-contained underwater breathing apparatus
2D: Two dimensional
HITS: High-intensity transient signals
EB scale: Eftedal–Brubakk scale for ultrasound bubble grading
BG: Bubble grade
BMI: Body mass index

## Code availability

Not available.
